# Expanding Understanding of Response Roles: An Examination of Immediate and First Responders in the United States

**DOI:** 10.3390/ijerph15030534

**Published:** 2018-03-16

**Authors:** Curtis Harris, Kelli McCarthy, E. Liang Liu, Kelly Klein, Raymond Swienton, Parker Prins, Tawny Waltz

**Affiliations:** 1 Institute for Disaster Management, College of Public Health, University of Georgia, Athens, GA 30602, USA; kmccarthy@uga.edu (K.M.); pprins@uga.edu (P.P.); twaltz@uga.edu (T.W.); 2 Department of Emergency Medicine, University of Texas Southwestern Medical Center, Dallas, TX 75390, USA; emberlynn.liu@utsouthwestern.edu (E.L.L.); kelly.klein@utsouthwestern.edu (K.K.); raymond.swienton@utsouthwestern.edu (R.S.)

**Keywords:** immediate responder, first responder, education, disaster response, community resilience

## Abstract

2017 was a record year for disasters and disaster response in the U.S. Redefining and differentiating key response roles like “immediate responders” and “first responders” is critical. Traditional first responders are not and cannot remain the only cadre of expected lifesavers following a mass casualty event. The authors argue that the U.S. needs to expand its understanding of response roles to include that of the immediate responders, or those individuals who find themselves at the incident scene and are able to assist others. Through universal training and education of the citizenry, the U.S. has the opportunity increase overall disaster resiliency and community outcomes following large-scale disasters. Such education could easily be incorporated into high school curriculums or other required educational experiences in order to provide all persons with the knowledge, skills, and basic abilities needed to save lives immediately following a disaster.

## 1. Introduction

The National Research Council’s Committee on Increasing National Resilience to Hazards and Disasters recommended in 2012 that increasing national level resilience should be a national priority [1]. In 2017, the U.S. experienced three major hurricanes, torrential flooding, rampant wildfires, two of the deadliest mass shooting events to date, and a number of other major disasters [2,3]. 2017 currently holds dubious distinction of being a record setter with sixteen “billion dollar disasters” in a single calendar year totaling more than $306 billion in damages [4]. The events of 2017 alone lend credence to the notion that increased disaster resilience is still needed throughout the U.S. This need to increase national resilience is not only due to both an escalation in overall disaster losses and increased frequency of disastrous events, but also due to a dearth of national capability to react to these events [1].

Responding to disasters can be extraordinarily complicated due to variations in which they manifest [5]. Whether natural, human systems failures, or intentional incidents, each disaster possesses unique and ubiquitous challenges to the overall response. Far too often, the public views the responsibility of disaster relief as a stand-alone obligation of state and/or federal government. In reality, that is simply not the case. Regardless of the disaster typology, recent disaster response efforts, including that following Hurricane Harvey, have revealed that is it often local populations that are best positioned and suited to begin response in the immediate aftermath of a disaster [6]. A successful local response is not merely an issue of proximity, but rather the implication of sharing a collective knowledge of local infrastructure, people, politics, and general sense of community. Similar conclusions concerning the implications of a local, immediate response were drawn following observation of the life-saving bystander intervention on Boylston Street in the immediate aftermath of the 2013 Boston marathon bombings [7]. The need for local population life-saving skill training and equipping has been demonstrated and is a known, vital component of disaster risk reduction [8,9]. 

The authors postulate that there are three categories of people that manifest following a disastrous event: the injured casualties, the immediate responders, and the first responders. The injured casualties are a group that may or may not be able to assist themselves (e.g., self-aid or communicating for help) but are likely unable to offer assistance to others in need. The immediate responder is an unprompted group of people comprised of both casualties with minor injuries and uninjured others located in direct proximity to an incident site. First responders describe a broad group that encompasses a trained cohort whose arrival to the impacted site is purposed to locate and initiate life-saving care and evacuation to the injured casualties. This paper will focus on the latter two groups, the immediate responder and the first responder. 

Policy efforts intended to enable effective disaster risk reduction strategies must primarily encourage and enhance proactive behavior at a local level. Craig Fugate, former Administrator of the Federal Emergency Management Agency (FEMA), was a proponent of such philosophy. In what would become the hallmark of his FEMA tenure, Administrator Fugate introduced the Whole Community or Community Based Disaster Management approach in 2011 [10]. The central force behind his philosophy was an understanding of community needs and capabilities and increased individual and collective preparedness. If proper whole community planning approaches succeed, protective actions taken to reduce disaster risk will improve environmental, social, and economic conditions at the local level [11]. 

Empowering local responders has the potential to be a force multiplier and increase national resiliency as whole communities develop the knowledge, skills, and abilities to better prepare for, mitigate, respond to, and recover from disasters. Building community resilience is an important component of disaster risk reduction strategy. Our understanding and definition of community resilience is still evolving; however, multiple common core elements have been identified that may lend to a community resilience framework [12]. At its core, community resilience is the ability of a community to anticipate risk and utilize available resources to respond to, withstand, and recover from adverse situations [13]. With such knowledge in hand, the issue now becomes operationalization. How does the community mobilize itself to handle the immediate aftermath of a disaster? The qualitative paper will seek to define the roles and responsibilities of immediate and first responders and propose mechanisms for educating and training the public to function in their respective roles.

## 2. The Immediate Responder 

The immediate responder is not a term commonly recognized by the emergency management community but is one that plays a pivotal role in life-saving intervention, urban search and rescue, and overall disaster response. The authors define the immediate responder as those individuals that through no desire or fault of their own are thrust into disaster response simply by being physically located in the immediate disaster zone. These individuals are often not formally trained in disaster management or disaster medicine but rely on natural instincts and local knowledge to visualize problems and devise ad hoc emergency response protocols to solve these problems with immediately available resources. Most immediate responders understand local infrastructure, may have unique specific knowledge of the incident site, and will often have a deep, personal connection to the area.

When a disaster occurs, those in the immediate area have two options: to provide assistance or not. One could certainly make the argument that self-preservation and fear would drive many to either flee the scene or become an event spectator as anxiety and panic replace rational thought processes. In the 1960s, two researchers postulated the theory of the bystander effect [14,15]. This theory suggests that, due to the sufficient possibility of negative outcomes that could result from rendering aid, bystander intervention will be low. However, this theory has been countered by the scientific community and bystanders in many recent events [16,17,18,19,20].

Images of the 1995 Oklahoma City bombing, 2001 World Trade Center attacks, the 2013 Boston Marathon bombing, recent hurricanes in the U.S., and countless other worldwide natural, technological, and man-made events prominently display the efforts of bystander intervention. Studies by Amanda Ripley and Thomas Drabek suggest that panic among bystanders is rare and preventable [17,18]. They postulate that panic only occurs when people feel trapped, helpless, and isolated and that panic can be quickly overcome by clear communication. Additionally, Jacobs et al. reported that 75% of respondents in a nationally representative survey indicated that they would assist in providing bleeding control in a mass shooting scenario [20]. Furthermore, Bakke et al. found that in trauma cases where bystanders were available to administer care, 81% of patients experienced successful hemorrhage control prior to the arrival of first responders and definitive care at a hospital [19]. Subsequent findings also showed that bystanders with prior knowledge and training on bleeding control measures demonstrated greater proficiency while administering aid than did those without proper training. Based on these findings, coupled with the dramatic increase in mass casualty events from terror attacks and natural disasters, logic dictates the implementation of substantial training and education efforts for all citizens to improve health outcomes of those affected. The difficult part is how and when to implement such a widespread educational program. For the purposes of this article, the authors will address the issue of education and training from a U.S. perspective.

Under the Whole Community framework, all community stakeholders work together to understand and assess the needs of the community to determine mechanisms through which to strengthen their assets, capacities, and interests [10]. While this system has significant benefits and has been extremely successful in locations that adhere to its principles, there are limitations. One of the primary issues in emergency management is acquiring funding to put proactive measures in place prior to a potential disaster. Instead, funds are often only allocated post-event to assist in response and recovery. It is also difficult to develop education and training to be disseminated to the masses that is scientifically validated and rigorously tested at the community-level. The federal government and private sector have developed publicly available, online educational programs and training classes to circumvent this issue. However, these courses often lack regional specificity and can be very technical leading to limited comprehension and retention. There are some online and in-person resources specifically targeted at immediate responders providing aid in mass casualty and/or trauma situations. These resources include Stop the Bleed, Until Help Arrives, Bleedingcontrol.org, and Bleeding Control for the Injured. The issue with these courses is that the normal citizenry either does not know the resources exist or fails to comprehend their own need to take such a course. The challenge therefore becomes how the emergency management community can ensure that this information effectively reaches the masses.

Israel, a world leader in disaster and emergency management and medicine, developed an ingenious, if perhaps accidental, solution to this issue. The State of Israel has a mandatory military service for all of its citizens. It is the only country in the world that maintains obligatory service for women [21]. It should be noted that there are exceptions to the mandate, but approximately 80% of Israeli citizens serve. By serving in the military, Israeli citizens receive requisite training in combat care and field treatment that make them ideal immediate responders if and when an attack occurs. The authors are by no means advocating that the U.S. implement this mandate on its citizens but it does point to a mechanism of conducting training where there is required citizen attendance.

In the U.S., the only place where a similar return on educational investment could be seen would be in high school curriculum [22]. A precedent has been set for implementing such programs into high schools. By 2017, 32 states passed legislation requiring high school students to be trained in cardiopulmonary resuscitation and automatic electric defibrillators prior to graduation [23,24,25]. There is absolutely no reason why Stop the Bleed, airway management, or other similar programs/principles could not be added to this curriculum or integrated into existing health, physical education, or similarly required courses. By incorporating this program of study into high schools, the U.S. would dramatically increase its responder network with trained individuals rather than people relying on instinct. It is likely that the number of lives saved would increase and people would be more confident in their abilities and presumably, willingness to help.

## 3. The First Responder 

Upon initial consideration of the term first responder, images of lights and sirens often jump to the forefront. It has been engrained in the U.S. culture that first responders are police officers, firefighters, and emergency medical technicians, etc. However, in the emergency management and medicine community, the term is now taking on differing connotations and its definition is expanding. As Craig Fugate so elegantly stated, “When you step back and look at most disasters, you talk about first responders—lights and sirens—that’s bullshit. The first responders are neighbors, bystanders, and the people that are willing to act.” [26]. The authors differ slightly with the former FEMA Administrator’s assessment of first responders, as there are times when neighbors and bystanders will be immediate responders rather than first responders, but will use his words to redefine the term. First responders are educated and trained individuals, organizations, and agencies that make a conscious decision to mobilize from an unaffected or lesser-affected area to the affected area (scene) in order to render assistance and aid. The Merriam–Webster dictionary defines that first responders are responsible for going immediately to the scene to provide assistance [27]. This is a difficult statement to make in the context of disasters because immediate can have differing definitions. It could mean immediately after the disaster has subsided or immediately after reasonable access to the area has been granted.

Large-scale disasters and catastrophic events differ significantly from everyday emergencies with regards to resource and manpower needs. In the aftermath of hurricane Harvey, FEMA Administrator Brock Long called upon the media to assist the government response by organizing citizen efforts to help Texas as needs exceeded available government resources [28]. The nation responded. From as far away as Los Angeles and Boston, first responders descended upon Texas. The Louisiana Department of Wildlife, the Cajun Navy, DieselSellerz.com, and countless men and women mobilized trucks and flat-bottom boats for ad hoc search and rescue efforts. Faith based organizations, private companies, and non-governmental organizations opened shelters and provided food for victims. J.J. Watt, a professional football player for the Houston Texans, started a fundraiser for hurricane Harvey relief that exceeded $37 million from more than 200,000 donors worldwide [29]. Even though the anecdotes listed above are from a single event, countless other examples exist of ordinary people, organizations, and agencies becoming first responders. While augmenting responder efforts is a critical need following a disaster, it is not without its problems.

Despite good intentions, spontaneous volunteerism and convergence can be a detriment to an overall response if not properly coordinated. Challenges can present in coordination, integration, communication, logistics, and health and safety [30]. When people, organizations, and agencies become involved in response efforts it is essential that they know their role(s), complete tasks given to them, report to leadership for new assignments upon completion of previous tasks, and work within the outlined framework of the response. Just one agency/responder going rouge can be a huge impediment for the overall response. The most effective way to circumvent these issues is via education and training. The good news is that educating first responders should be somewhat easier than educating the immediate responders due to their desire to be involved and the likelihood that they belong to an established, formal organization of responders.

There are a number of education and training opportunities available in-person and online for training first responders. It is important to note that these individuals need training not only in basic lifesaving interventions, search and rescue, resource allocation, etc., but also in the fundamental principles of incident command, the National Incident Management System (NIMS), event management, and so on. Training in the U.S. for each of these areas can be obtained via FEMA online certifications, Community Emergency Response Teams, Medical Reserve Corps, American Red Cross, facility specific education and training, and many others. 

However, it is not enough to simply receive education and training. Additional knowledge, skills, and abilities must be tested and practiced in ongoing drills and exercises to ensure true understanding of roles and responsibilities in disaster response. Perhaps the most advantageous way to become involved in exercises and be incorporated into the disaster response framework is through involvement in healthcare coalitions. Healthcare coalitions are made up of healthcare organizations, public safety officials, public health partners, private businesses, non-governmental organizations, and private citizens that work together to ensure communities are healthier, safer, and more resilient [31]. The beauty of coalitions is that they allow the emergency management community to pool together limited resources to conduct exercises and training while also being multidisciplinary and allowing for plan integration [31]. The latter will prove to be extremely important as evidenced by the National Security Strategy outline by the Trump administration [32]. 

President Trump released his National Security Strategy on 18 December 2017. In the “Promote American Resilience” section of the document, one of the primary actions is to build a culture of preparedness by informing and empowering communities and individuals to obtain the skills necessary to become more resilient against threats and hazards. This strategy is in direct alignment with what FEMA Administrator Brock Long has been promulgating throughout the devastating hurricane season of 2017—state and local governments must be able to sustain themselves and that FEMA was not, and was never meant to be, a first responder organization [33]. Based on this assertion, there is a simple correlation that can be made to an old adage, it not only “takes a village to raise a child” but also takes a village to respond to a disaster.

## 4. Conclusions

The premises upon which disaster risk reduction and building community resilience are achieved, begin with strengthening and empowering an equipped local citizenry with education, skills and training. Understanding the expansion of current responder definitions is critical for disaster response and community resilience. Immediate responders are often acting as individuals while their first responder counterparts are often more organized and have established and understood chains of command, protocols, etc. This does not mean, however, that the two groups are always mutually exclusive. For example, someone who received immediate responder training in a basic high school health course may eventually also take more courses and earn additional training to move into that first responder role. Likewise, a physician who happens to be at the initial site of a bombing may begin applying improvised tourniquets and pressure dressings at the scene (in the role of an immediate responder) but later be integrated in a more formalized incident command structure and continue in-field treatments in a more formal first responder capacity. Furthermore, while some targeted educational programs exist and training materials are available, the challenge to improving the desired resiliency is in the accessibility of said materials. Incorporating core concepts into a required high school level curriculum would be a way to ensure information is universally available to the targeted populations.
